# Integrated network analysis reveals new genes suggesting COVID-19 chronic effects and treatment

**DOI:** 10.1093/bib/bbaa417

**Published:** 2021-02-11

**Authors:** Alisa Pavel, Giusy del Giudice, Antonio Federico, Antonio Di Lieto, Pia A S Kinaret, Angela Serra, Dario Greco

**Affiliations:** Faculty of Medicine and Health Technology, Tampere University, Tampere, Finland; BioMediTech Institute, Tampere University, Tampere, Finland; Faculty of Medicine and Health Technology, Tampere University, Tampere, Finland; BioMediTech Institute, Tampere University, Tampere, Finland; Faculty of Medicine and Health Technology, Tampere University, Tampere, Finland; BioMediTech Institute, Tampere University, Tampere, Finland; Department of Forensic Psychiatry, Aarhus University, Aarhus, Denmark; Institute of Biotechnology, University of Helsinki, Helsinki, Finland; Faculty of Medicine and Health Technology, Tampere University, Tampere, Finland; BioMediTech Institute, Tampere University, Tampere, Finland; Faculty of Medicine and Health Technology, Tampere University, Tampere, Finland; BioMediTech Institute, Tampere University, Tampere, Finland; Institute of Biotechnology, University of Helsinki, Helsinki, Finland

**Keywords:** COVID-19, SARS-CoV-2, coronavirus, virus–host interaction, unified knowledge space, data integration, multi-layer network analysis, drug targeting, drug repositioning

## Abstract

The COVID-19 disease led to an unprecedented health emergency, still ongoing worldwide. Given the lack of a vaccine or a clear therapeutic strategy to counteract the infection as well as its secondary effects, there is currently a pressing need to generate new insights into the SARS-CoV-2 induced host response. Biomedical data can help to investigate new aspects of the COVID-19 pathogenesis, but source heterogeneity represents a major drawback and limitation. In this work, we applied data integration methods to develop a Unified Knowledge Space (UKS) and used it to identify a new set of genes associated with SARS-CoV-2 host response, both in vitro and in vivo. Functional analysis of these genes reveals possible long-term systemic effects of the infection, such as vascular remodelling and fibrosis. Finally, we identified a set of potentially relevant drugs targeting proteins involved in multiple steps of the host response to the virus.

## Introduction

The newly identified coronavirus SARS-CoV-2 is responsible for a pandemic form of respiratory tract infection currently ongoing worldwide. Even if most patients remain asymptomatic or show mild symptoms, some develop complications, such as severe pneumonia and acute respiratory distress syndrome (ARDS) [1, 2]. Furthermore, systemic complications, such as cardiovascular disorders, persistent lung injuries and possibly fibrosis are rapidly emerging as key threats in addition to the respiratory syndrome. Restrictive measures have been adopted to slow down the spreading of the virus; however, it is expected that the infection will remain entrenched in the population for years [3].

To date, no approved vaccine is yet available and some therapeutic strategies have been proposed to control the clinical outcomes of the infection [4, 5]. Currently, a great effort is being made by the scientific community in order to develop new therapeutic approaches as well as to understand the molecular events characterizing the host response to SARS-CoV-2 infection. SARS-CoV-2 infects the cells via the angiotensin converting enzyme 2 (*ACE2*) receptor-mediated endocytosis [6]. *ACE2* is expressed in several organs and cell types, such as lung, heart, kidney, intestine and endothelial cells, which further raises concerns about possible ectopic effects of the infection [7].

Molecular characterization of infected tissues and cells can elucidate key potential molecular targets involved in the pathogenesis of COVID-19. To this end, for instance, Gordon *et al*. [8] applied mass spectrometry to identify SARS-CoV-2 human protein interactors. These proteins can be considered as the first responders to the virus, acting upstream in the host response to the infection. Moreover, transcriptomic data of infected lungs and cell types are already publicly available [9]. On the contrary, the genes derived from the transcriptomic data represent late effectors in the host immune response. Nonetheless, a knowledge gap exists to link the first host responses to the virus with the subsequent phenotypic alterations. In this work, we hypothesize that genes linking the upstream interactors and downstream effectors are involved in the transduction and amplification of the host response to the virus and can therefore represent a new set of potentially relevant genes. Developing computational methods that are able to infer such missing information is of extreme importance, especially in situations where there are limited data available, such as in the COVID-19 disease. Moreover, a deeper understanding of the underlying molecular responses is required in order to develop suitable treatment methods and prepare for possible long-term effects. This gap could be filled by exploiting the large amount of biomedical data accumulated in recent years. However, the use of this information is currently hampered by the heterogeneity of data formats scattered across multiple repositories [10–12]. In this study, we applied scalable and flexible data integration methods to develop a robust compendium of molecular knowledge, the Unified Knowledge Space (UKS). Knowledge graphs (KGs) are large data structures that model different entities, their properties and relationships [13–16]. KGs allow to integrate multiple data from diverse domains and repositories into a common space. In this way, KGs facilitate the organization of information in a structured manner and allow to visualize and retrieve complex relationships between different entities derived from multiple sources. Another purpose of KGs is the generation of currently unknown facts, which can be inferred from existing links in the KG. In the domain of biology, KGs have for example been used in drug repositioning [17, 18] or to infer disease-biomolecule associations [19, 20]. In our UKS, nodes can be genes, gene products or drugs, while edges represent different relationships between the entities. The UKS is created by combining homogeneous with heterogeneous network integration methods. Homogeneous network integration combines different networks with the same node (type) but different edges, merging them into a single network (e.g. combining multiple protein–protein interactions (PPI) networks), while heterogeneous network integration aims at connecting networks with different node (types) (e.g. gene–drug target networks with a gene–gene network) [21].

The expansion of the PPI network through other data types to construct a heterogeneous network has been previously applied in a variety of contexts [20, 22, 23]; Davis and Chawla [23] constructed a phenotypic-disease network merged with a genetic-disease network to investigate disease comorbidities, while Goh *et al*. [20] built a network linking genetic disorders with known disease genes to investigate the role of disease genes in the human interactome. A detailed review about different network data integration methods and their application is provided by Gligorijević and Pržulj [21]. While previous studies aimed at constructing a homogeneous or heterogeneous network for a specific case study, we built an expandable and flexible data structure. Consequently, high-quality networks can be inferred (homogeneous and/or heterogeneous networks can be retrieved). This allows the UKS to be used in a wide variety of different studies in the future.

We analysed the UKS and retrieved a novel set of genes potentially associated with the molecular host response to SARS-CoV-2 infection. The functional characterization of this new set of genes allows us to describe possible unpredicted long-term complications of the COVID-19 disease, as well as to suggest repositioning of some already approved drugs.

## Methods

The proposed methodology aims at giving insights into the possible mechanistic aspects of the SARS-CoV-2 infection and host response through the construction of a Unified Knowledge Space. Combining knowledge about viral physical interactor human proteins and transcriptomic studies into a single knowledge space allows to gain new valuable insights about the mechanisms underlying COVID-19. By further expanding the UKS with information about drug targets, valuable novel knowledge regarding multiple facets of the SARS-CoV-2 infection can be generated. We define the UKS as a knowledge graph constituted through multiple network layers [24, 25], where nodes are representing either gene (products) or drugs, and edges represent either direct known physical gene–gene interactions or drug–gene target relationships. The UKS comprises all human protein-coding genes as retrieved from Ensembl [26], known physical interactions of their associated proteins as well as all known drug target relationships. Our whole applied pipeline, including data retrieval, processing and knowledge extracted, is outlined as pseudocode in the Supplementary File S1 available online at https://academic.oup.com/bib.

## Data collection

### Viral interactors

Genes known to be physically interacting with the viral components of SARS-CoV-2 were retrieved from [8]. These genes are involved in the first events of the host response upon viral infection.

### Transcriptomics data

The gene expression data of human lung biopsies of SARS-CoV-2 infected patients and SARS-CoV-2 infected cell lines were retrieved from the Gene Expression Omnibus (GEO) repository (GEO ID GSE147507) [9]. The dataset only consisted of one time point, and RNA was extracted 24 h after the infection. In this work, we analyzed five different experimental conditions contained in the GEO dataset: human lung biopsies of SARS-CoV-2 infected patients and uninfected control; A549 cell line infected with SARS-CoV-2; A549 cell line infected with SARS-CoV-2 overexpressing ACE2; Calu-3 cells infected with SARS-CoV-2; NHBE cell line infected with SARS-CoV-2. For each of the cell lines, the mock treated lines were collected to be used as controls for the expression analysis.

### Transcriptomics data analysis (DE gene set identification)

Gene expression analysis was carried out starting from the raw counts provided within the GEO record. Low read counts were filtered by applying the proportion test method implemented within the NOISeq Bioconductor package [27]. Filtered counts were normalized through the upper quartile method implemented in the NOISeq package. Differential expression analysis was carried out by using the DESeq2 Bioconductor package [28], while p-values were adjusted through the Benjamini-Hochberg method [29]. The pre-processed expression matrices are reported in the Supplementary Files S2–S5 available online at https://academic.oup.com/bib.

## UKS construction and PPI network retrieval

Known human protein coding genes were retrieved from Ensembl (Assembly: GRCh38) [26], which represent the base of the developed UKS. Known protein–protein interactions were retrieved from HIPPIE (downloaded 28/10/2019) [30], HitPredict [31, 32] (downloaded 04/11/2019), KEGG (downloaded 08/12/2019) [33] and STRING (downloaded 23/02/2020) [34]. We combined these PPI networks into a unique homogeneous network, by mapping the proteins to their associated genes. The edges were weighted based on an interaction source support score, where an edge weight of 1 indicates source support by 100% of the collected sources. This is important, since it has been shown that there is a high variance between links in PPI networks, in terms of quality of the determined interactions (e.g. experimental based versus literature based). Therefore, the confidence of the interaction varies widely and, in addition, links between proteins may be missing [35–37]. To reduce the data quality bias, we consider source support for each edge as important to reveal a high confidence subnetwork from the homogeneously merged PPI network. This approach is similar to the robust PPI network construction approach suggested by Martha *et al*. [38]. Drug target information was collected from DrugBank (downloaded 22/04/2020) [39] and Open Targets (downloaded 15/02/2019) [40] and integrated into the UKS. The data contained in these two sources are merged into a single data layer by means of mapping drugs to PubChem CIDs or SIDs [41], again conserving source information. In order to link the data accurately to the previously discussed data layers, gene symbols are mapped to Ensembl Gene IDs [26] through mygene.info (http://mygene.info) [42, 43]. To provide a highly flexible data provisioning system, the UKS is stored as a graph database in Neo4j 4.0 (https://neo4j.com/), which allows to edit, retrieve and add new data as needed. The complete UKS contains 20 793 human protein coding genes, which are interlinked by 5 941 639 edges, representing physical known interactions. Additional 7099 drugs are linked through 22 973 edges to their genetic targets. To construct a high-quality gene–gene network, gene–gene relationships, associated with a source support score of at least 0.75, are queried from the UKS and used to construct a single layer gene–gene network, which is represented as a Python NetworkX graph [44]. The final gene–gene network is made up of 20 793 nodes, representing Ensembl gene IDs, interlinked by 132 244 high-quality edges, describing interactions between the gene’s associated proteins.

### Identification of intermediate genes through shortest paths

In order to identify the relevant genes that may have a crucial role in the progression of SARS-CoV-2 infection, the shortest paths between the physical interacting (PI) and the differentially expressed (DE) gene sets were computed. Shortest path analysis is a method to link two sets of nodes of interest and identify interactor nodes between them. On a graph *G* = (*V*, *E*), where *V* is the set of nodes and *E* is the set of edges, a shortest path between vi and vj is defined as the path between vi and vj requiring the least effort. In an unweighted network, this translates into the least number of steps to be taken to connect vj and vi [45]. The shortest path analysis was performed for each group of DE genes identified in each biological system.

The shortest paths were retrieved with Python NetworkX [44], shortest_path() function, by running Dijkstra’s shortest path algorithm [45] between all possible pairs of PI and DE genes (Python 3.6.9, NetworkX 2.3). All edges were considered to have equal weight, meaning that only the number of steps was considered when running the algorithm. Only paths consisting of at least one intermediate gene (IN) (path length >1) were considered during further analysis.

For each gene in the PPI network, its occurrences as an IN between PI and DE was estimated separately for each experimental class and statistically significant enriched IN genes were identified. Hypergeometric test was performed by comparing the IN frequencies identified in the shortest paths of interest with their occurrences on all possible shortest paths in the complete gene–gene network. By estimating statistical significance of each visited intermediate node, only intermediate nodes that are relevant in linking the previously defined sets of key nodes (DE and PI) are considered. Adjusted p-values were estimated by applying the Benjamin and Hochberg multiple testing correction [29]. The nominal p-values were calculated with Python’s SciPy package [46] and the adjusted p-values were estimated based on Python’s statsmodels package [47] (SciPy 1.3.2, statsmodels 0.11.1).

### Pathway enrichment analysis

In order to functionally characterize the lists of PIs, INs, and DEs, pathway enrichment analyses were performed using the Wikipathway 2019 Human database through the EnrichR online tool [48, 49]. The enriched pathways were visualized by means of the FunMappOne tool [50].

### Gene ranking

In order to evaluate the overall most common genes crossed in the shortest paths, for each in vivo and in vitro system, only statistically significant genes were selected and ranked according to the intermediate gene count value. The five lists were given as an input to the Borda function of the TopKList R package [51], to calculate the Borda scores and rank the genes according to the median function.

### Identification of relevant drugs

In order to highlight drugs that could simultaneously affect multiple steps of the host response to SARS-CoV-2, we retrieved from the UKS the list of drugs targeting genes in the PI, IN and DE sets and retrieved the set contained in their intersection.

## Results and discussion

### A novel set of genes involved in the pathogenesis of COVID-19 can be retrieved from multi-scale molecular network analysis

**Figure 1:**
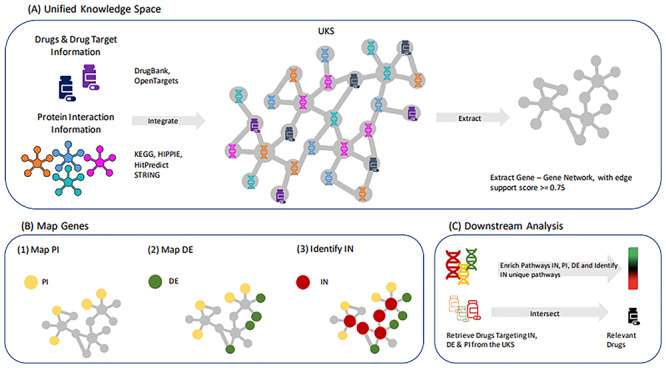
Scheme of the analytical framework. Data from multiple protein–protein interaction (PPI) sources (KEGG, HIPPIE, HitPredict and STRING) were collected and mapped to their corresponding Ensembl Gene IDs. The PPI network was further integrated with drug–target information, derived from DrugBank and OpenTargets, to form the UKS (**A**, left). A robust gene–gene network was extracted from the UKS, where only edges supported by at least three of the merged PPI networks were included (**A**, right). PI and DE genes were mapped onto the extracted gene–gene network and intermediate genes (IN) were identified by means of shortest paths between each possible pair of PI and DE (**B**). Pathway enrichment analysis was performed for all three gene sets. Drugs that have targets in all three gene sets (PI, DE and IN) were selected and classified as ‘relevant drugs’ (**C**).

The Unified knowledge space (UKS) defined in this work has been generated by integrating multiple data sets containing protein–protein interaction (PPI) information as well as drug–target relationships. By querying the UKS, we derived a network of 20 793 human protein coding genes, represented as nodes, and 132 244 edges, representing the physical interaction relationships existing between the proteins encoded by the UKS gene nodes. These interactions were integrated from four data sources and stored in the UKS together with a data support score, representing the number of sources in which the connections are present. In order to have a reliable structure of the network, we selected only edges supported in at least three out of four sources (see section ‘UKS Construction and PPI Network Retrieval’ for more details). The UKS network was further extended with gene–drug information by adding 7099 drug nodes that are linked to their target gene nodes through 22 973 edges (Figure 1A). We systematically mapped the SARS-CoV-2 physical interacting (PI) genes and the differentially expressed (DE) genes in multiple biological systems infected by SARS-CoV-2 [9] (Figure 1B).

A set of human proteins has been recently described by Gordon *et al.* as physical interactors of the SARS-CoV-2 viral components [8]. We considered these as the first set of proteins involved in the host response to a SARS-CoV-2 infection. On the other hand, we considered the differentially expressed genes retrieved from transcriptomic analysis of infected *in vivo* (infected versus healthy human lung biopsies) and *in vitro* (infected versus mock CALU-3, A549, A549 overexpressing *ACE2*, and NHBE cell lines) systems, as late effectors associated with the COVID-19 pathological phenotype. In order to identify the relationships between the first interactors of SARS-CoV-2 (PI gene set) and the late effectors (DE gene set), a third set of genes, located in the shortest path between each possible pair of (PI-DE genes) was retrieved.

The concept of shortest paths has already been widely applied in the analysis of biological networks and has yielded biologically relevant results [52–54]. Du *et al*. [52] mapped differentially expressed genes onto a PPI network and successfully identified transcription factors linking a cancer gene to its differentially expressed genes. Simões *et al*. [53] applied a similar strategy in order to identify genes associated to complex diseases.

In our study, we use the concept of shortest paths to investigate the set of genes linking the genes directly interacting with viral components and the ones whose transcription is altered by the induced host response. From a kinetics perspective, the first set of genes (PI) can be assumed to have a role in the first molecular events upon viral exposure; on the contrary, modulation of the expression of the late effector genes (DE) is associated with cellular and, ultimately, systemic response to the infection. We, therefore, assumed that genes in the shortest paths can be involved in the transduction and amplification of the host response. In this light, the intermediate genes can better explain the chain of the molecular events characterizing the response to SARS-CoV-2, as well as can represent another important set of therapeutic targets.

For each in vitro and in vivo system analyzed, we named as intermediate genes (IN gene set), all the genes, not belonging to either the PI nor the DE gene sets, significantly overrepresented (*P*-value ≤ 0.05) in the shortest paths.

**Figure 2:**
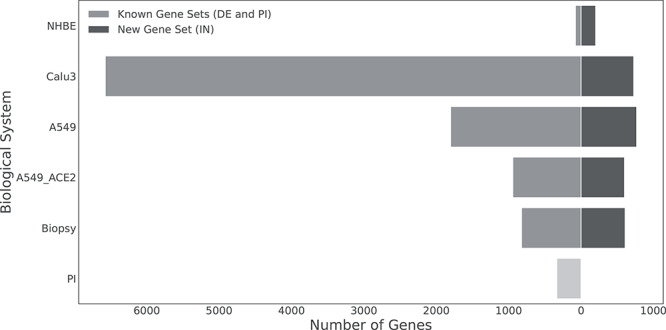
Number of known gene sets (DE and PI) and new gene set (IN) in each biological system. The number of differentially expressed genes (DE), and the new retrieved set of intermediate genes (IN), are compared in each *in vitro* and *in vivo* system. The samples (biological system) derived from public transcriptomics dataset comprising a lung biopsy and four different cell lines infected with the virus: the transformed cell lines A549 (adenocarcinomic human alveolar basal epithelial cells) and CALU-3 (human lung cancer epithelial cell), the epithelial cell line NHBE and A549 overexpressing the angiotensin receptor ACE2 (A549_ACE2).

In contrast with the heterogeneity of the DE gene set sizes, the number of intermediate genes is comparable among the different biological systems (Figure 2). Overall, we observe a progressive increase in the size of the gene sets when going from the first interactors (PI), through the intermediate genes (IN), to the effector pathways genes (DE), suggesting the role of the intermediate genes in propagating the host response mechanisms to the virus entry. The human bronchial epithelial cells (NHBE), on the contrary, was the only dataset showing a decreasing trend from the PI to the DE gene set. This is probably due to the smaller number of differentially expressed genes, which can be associated with the lower permissiveness of the NHBE cell line.

### Functional characterization of intermediate gene set reveals possible long-term effects of COVID-19 disease

**Figure 3:**
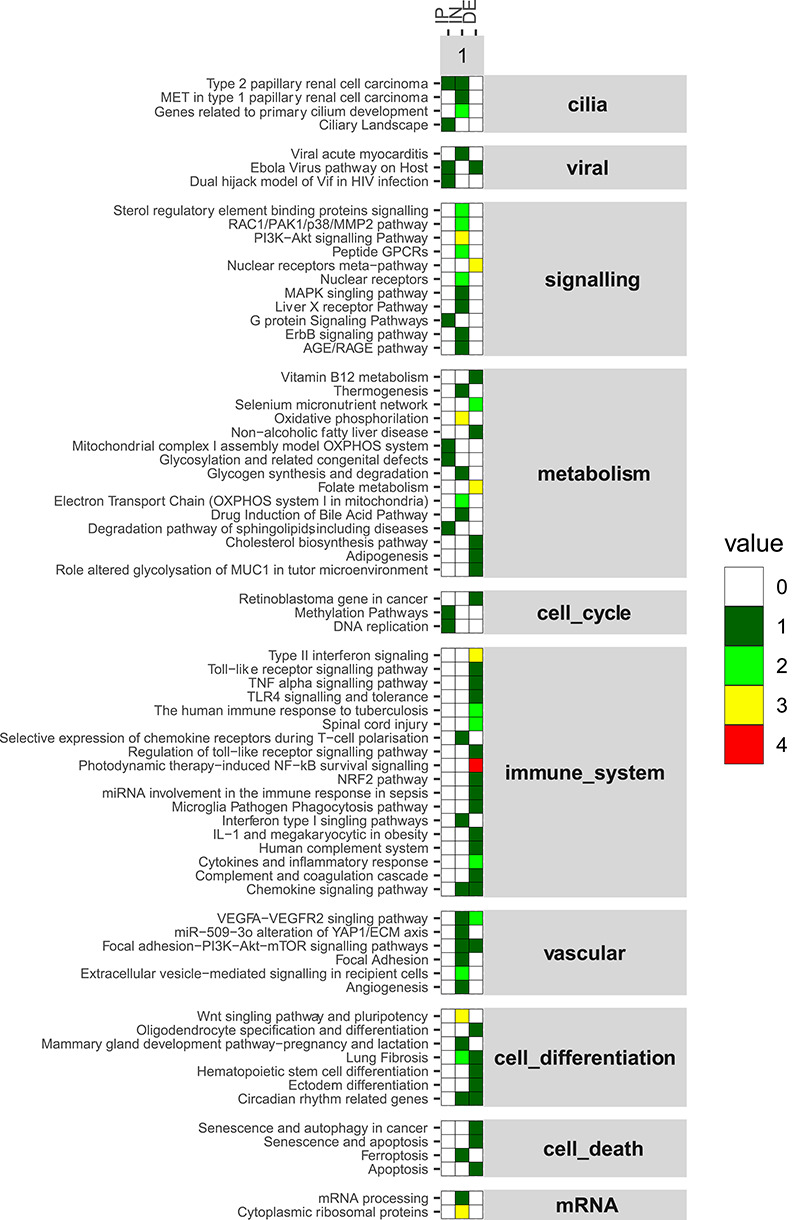
Pathway enrichment of the PI, IN and DE gene sets. For each biological system (in vitro and in vivo), significantly enriched Wikipathways by the three sets of genes (PI, IN, DE in the columns) are shown (rows). The number of samples that enriched specific pathways are marked with different colours (values). Furthermore, the enriched pathways have been grouped according to more generic biological processes (cell differentiation, cell metabolism, cell death, metabolism, immune system) or molecules and structures (mRNA, viral, vascular and cilia).

In order to characterize the IN gene set, we performed pathway enrichment analysis independently for each in vivo and in vitro biological system analyzed. Moreover, we compared the pathways over-represented in the IN set with the ones over-represented in the PI and DE genes, respectively, in order to identify specific biological functions, which could fill the gap between the early molecular interaction events and the downstream transcriptomic host response (Figure 3).

As expected, PI genes specifically enriched pathways related to viral infections, such as Ebola Virus pathway on Host and Dual hijack model of Vif in HIV infection. Not surprisingly, cilia associated pathways were also enriched, since epithelial cells are the first ones to encounter SARS-CoV-2 in the respiratory system. These pathways are also well represented in the IN genes, while they are not significantly enriched in the DE gene set.

Metabolic pathways are present in all the gene sets (PI, IN and DE), with the oxidative phosphorylation and lipid metabolism being the most affected functions. Viral infections are known to induce a global metabolic alteration of the cell and, in particular, lipids play a pivotal role in facilitating viral replication [55].

DE genes specifically enriched immune system related pathways, which were not represented in the PI gene set and minorly represented in the IN set. Some of the main effector molecules involved in the cytokine storm observed in COVID-19 were present in the enriched immune pathways (e.g. INFγ, TNF, IL-1β and other chemokines) as well as the NFkB transcription factor pathway (Figure 3) [6, 56]. Interestingly, interferon response was retrieved as significantly over-represented both in IN and DE genes. However, type I interferon was specifically enriched in the IN set, whereas type II was enriched in the DE set only. Interferon gamma, the only type II interferon, is one of the genes involved in the cytokine storm [56]. On the contrary, type I interferons are key antiviral mediators, and low levels have been described in COVID-19 patients [57] .

Both IN and DE genes enriched pathways related to cell differentiation, such as lung fibrosis, Wnt pathway and ectoderm differentiation. As we already reported, COVID-19 disease shares many mediators of the lung fibrosis pathogenesis, such as *NFkB*, *IL-6*, *TGF* and *INF* [58]. Furthermore, the receptor ACE2 is a known anti-fibrotic mediator, and lung fibrosis has already been reported subsequently to the outbreak of SARS-CoV [59], making it also a plausible long-term consequence of SARS-CoV-2 viral infection. IN genes specifically enriched the Wnt pathway, which has been linked to chronic lung pathologies, including idiopathic pulmonary fibrosis, pulmonary arterial hypertension, asthma and chronic obstructive pulmonary disease [60]. Altogether, this suggests that fibrogenic alterations in the lung can be a possible long-term effect of the COVID-19 pathogenesis, as we have already recently suggested [58].

Finally, the IN gene set enriched specific biological functions represented in neither PI nor DE. Signalling related pathways, with the exception of nuclear receptors, are only present in the IN group. This indicates the central role of the IN genes in propagating the signal from the PI initial interactors to the late effector pathways. Consistently, mRNA processing pathways are only enriched in the IN group.

Therefore, the pathway enrichment of the newly identified IN set of genes reveals specific categories that represent signalling and metabolic pathways. These intermediate pathways are filling the gap between the first interactors and the late effector pathways, as well as cell differentiation pathways, suggesting possible long-term lung tissue remodelling.

### The intermediate genes are also linked to endothelial cells dysfunction and vascular remodelling

Interestingly, the IN gene set also enriched vascular related pathways. Among them, we found VEGF signaling pathways, angiogenesis, EPO signaling and extracellular matrix related pathways. Ackermann *et al.* recently showed that lung tissue of SARS-CoV-2 infected patients presented endothelial damage and significant new vessel growth [61]. The overall modulation of vascular related pathways highlighted in the IN genes, as well as the previously described cell differentiation pathways, may be an indication of endothelial remodelling and dysfunction. Endothelial dysfunction refers to a systemic condition in which the endothelium loses its physiological properties, including the tendency to promote vasodilation, fibrinolysis and platelets aggregation [62]. Different studies already proposed the endothelium as one of the main targets of SARS-CoV-2 [63–65], furthermore increasing evidence of coagulation alterations and fibrotic lesions are currently emerging in the scientific literature [63, 66]. Therefore, the new set of IN genes further strengthens the notion that the endothelial cells play a pivotal role in the COVID-19 disease and can help in predicting long-term effects in the lung in terms of vascular remodeling and dysfunction.

We further compiled five ranked lists of intermediate genes (for each in vitro and in vivo system represented in the DE space), according to the frequency in which they occurred in the list of shortest paths identified in each biological system. To obtain a final consensus rank, we merged the lists by using the Borda method (Supplementary File S6 available online at https://academic.oup.com/bib).

Leucine-rich repeat kinase 2 (*LRRK2*) is the most frequently visited gene in the shortest paths identified in the gene–gene network retrieved from the UKS. This gene, which has been extensively studied for its role in Parkinson disease [67], is known to upregulate the transcriptional activity of *NFkB* by increasing phosphorylation levels of *NFkB* inhibitor alpha (*IkBa*). Hongge *et al.* proposed that *LRRK2* has the potential to be an important target for the treatment of endothelial dysfunction [68]. Furthermore, Marker *et al.* [69] demonstrated that in HIV infection, *LRRK2* decreases the levels of the angiogenesis inhibitor BAI1 and increases the production of pro-inflammatory cytokines and phagocytosis. Given the pivotal role of *NFkB* in the COVID-19 disease, *LRRK2* is potentially important in both acute and long-term responses.

*Cullin 3* (*CUL3*), the third gene in the rank, has a role in endothelial remodelling and angiogenesis, both in physiological and pathological conditions [70].

The Exportin 1 (*XPO1*) gene is known to modulate the activity of mothers against decapentaplegic homolog 3 (*SMAD3*), a well-established initiator of epithelial mesenchymal transition (EMT) [71]. *SMAD3* is an important downstream transcription factor of TGF-beta, which regulates the transcription of extracellular matrix components involved in cellular infection [72]. Interestingly, XPO1, together with SMAD3 and TGF-beta, are strongly linked to lung fibrosis [73, 74]. Similarly, heat shock protein family A (*Hsp70*) member 4 (HSPA4), a chaperone protein, modulates the expression of transcription factor *TWIST1*, a master regulator of morphogenesis and epithelial mesenchymal transition [75].

The histone variant *H2AX*, a sensitive marker of DNA repair machinery, is also present among the top genes of the Borda ranking. There is evidence that it plays an important role in endothelial cell proliferation under hypoxia and, more generally, in hypoxia-induced angiogenesis.

The heterogeneous nuclear ribonucleoprotein A1 (*hnRNPA1*) gene has the capability of controlling migration, proliferation and gene expression levels of vascular smooth muscle cells. A recent functional study showed that not only *hnRNPA1* is an important regulator in vascular smooth muscle cells function and lesion-induced vessel remodeling but may also represent a potential therapeutic target [76].

Finally, during lung epithelium infection, an important role in activating both the innate and adaptive immune system and the tissue repair mechanisms is also played by the estrogen receptors [77]. Furthermore, anti-inflammatory effects of estrogens have already been reported [78] and are also supported by our results since both estrogen receptors *ESR1* and *ESR2* are contained in the top ranked genes (Supplementary File S6 available online at https://academic.oup.com/bib). Based on our results, our novel UKS is able to highlight key genes involved in possible long-term effects of SARS-CoV-2, which are associated with vascular remodelling and endothelial dysfunction, and in some cases have already been pointed out as interesting therapeutic targets.

### Drugs targeting genes in all gene sets suggest repositioning of drugs with anti-angiogenic and immuno-modulatory properties

Given the functional importance of the IN gene set, we further investigated whether these genes could also be molecular targets of known drugs. We retrieved information about drugs targeting the PI, IN and DE gene sets from the UKS.

**Figure 4:**
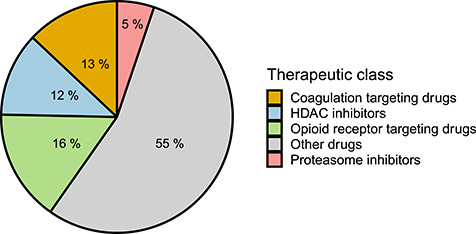
Overview of the 77 drugs targeting genes in all gene sets (PI, IN, DE). The list of 77 drugs sharing at least one target in each set of genes (PI, IN, DE) belong to four main therapeutic classes showing both immunomodulatory and anti-angiogenic properties: HDAC inhibitors (12%), proteasome inhibitors (5%), drugs targeting the opioids receptors (16%) and the coagulation cascade (13%).

Highlighting drugs, which can simultaneously target multiple components of the host response (PI, IN and DE gene sets) allows to uncover possible therapeutic strategies, which can more effectively reduce the clinical consequences of the viral infection [79, 80]. We hence identified 77 drugs targeting genes in all the three gene sets of interest (Figure 4 and Supplementary File S7 available online at https://academic.oup.com/bib).

Among the 77 drugs, four different therapeutic classes were strongly represented: HDAC inhibitors, proteasome inhibitors, drugs targeting the coagulation cascade and drugs targeting the opioids receptors (Figure 4).

Moreover, we found cough suppressants, such as dextromethorphan, hydrocodone and pentoxyverine, as well as expectorants and bronchodilators, such as theophylline, aminophylline and oxtriphylline [81]. These drugs are all centrally acting agents, thus exerting their effect on the lungs by inhibiting the cough centre in the brain.

Other well-represented drug categories were analgesics, antipsychotics and opioid antagonists. Haloperidol, amitriptyline, pentazocine and naltrexone, among others, belong to such categories. These drugs, together with the previously described dextromethorphan, hydrocodone and pentoxyverine, share the same molecular targets both in the PI and IN gene sets: the sigma non-opioid intracellular receptor 1 (*SIGMAR1*) and the μ opioid receptor (*OPRM1*), respectively (Supplementary File S7 available online at https://academic.oup.com/bib). Opioid drugs have a well-recognized effect on immune cells both modulating the immune system and exerting anti-inflammatory properties [82]. Besides, existing literature suggests that opioids might be able to interact with viral receptors, viral proteins, viral promoters and even modulate epigenetic mechanisms, such as the expression of anti-viral miRNAs [83]. In fact, dextromethorphan was already reported by Gordon *et al.*, because of its antiviral properties. On the other hand, dextromethorphan also shows immunomodulatory effects by decreasing *NF-*κ*B* and the *MAPK* cascade genes activation in LPS-treated dendritic cells, and interfering with primary T-cell responses [84]. On the contrary, naltrexone, an antagonist of the μ receptor, has been shown to revert the immunomodulatory action of opioids in several experimental models [85]. Since the sigma receptors have negligible affinity for naltrexone, it might be speculated that a significant part of the effect is exerted via direct binding to the opioid receptors. Taken together, these data suggest that compounds acting on the sigma opioid receptors might be involved in the innate and adaptive immunity in response to a SARS-CoV-2 infection and that they can have an effect in modulating the cytokine storm observed in the most severe and life-threatening stages of the disease.

Fostamatinib, a tyrosine kinase inhibitor, is also present in the list of identified drugs and importantly it targets *LRRK2*, the most commonly crossed IN genes in the shortest paths derived from the UKS. Fostamatinib is currently used to treat autoimmune diseases and thrombocytopenia, but it has recently been proposed for COVID-19 disease treatment by Saha *et al.* [86]. Similar to fostamatinib, we retrieved several drugs targeting the coagulation cascade, such as kappadione, a vitamin K analogue, and menadione, used in hypoprothrombinemia treatment. It has already been shown that COVID-19 patients commonly show thrombocytopenia and are at risk of developing disseminated intravascular coagulation, even though the molecular mechanisms have been poorly described [87, 88]. Thrombocytopenia is usually associated with an excessive activation of platelets and of the coagulation cascade, which can be triggered upon viral infection. Indeed, viruses have the ability of altering the balance between procoagulant and anticoagulant homeostatic mechanisms, as well as to induce pathogenic processes such as endothelial dysfunction, Toll-like receptor activation and tissue factor pathway inhibitor activation [87, 89].

Noteworthy, the drugs listed in Supplementary File S7 available online at https://academic.oup.com/bib highlighted possible repositioning of HDAC inhibitors. HDAC inhibitors are a class of compounds that act on epigenetic regulation of gene expression by increasing the lysine acetylation of histones [90]. They have antiviral properties by controlling the virus replication cycle and exerting cytotoxic activity, but they also have immunomodulatory properties by regulating the production of cytokines as well as the activity of macrophages and dendritic cells [91, 92]. Gordon *et al.* [8] showed that the SARS-CoV-2 non-structural protein 5 (Nsp5) interacts with the histone deacetylases and proposed valproic acid as a therapeutic agent in COVID-19. Our UKS system was able to detect several HDAC inhibitors, which target genes in all the PI, IN and DE sets: romidepsin, belinostat, entinostat, tacedinaline, fimepinostat, panobinostat, Cucd-101 and the valproic acid itself. Specifically, the eight HDAC inhibitors targeted the *HDAC2* gene present in the PI set, and the *HDAC5*, *HDAC7* and *HDAC11* present in the IN gene list, and *HDAC9*, *HDAC1*, *HDAC10*, *HDAC3*, *HDAC6* and *HDAC8* in the DE set. *HDAC2* is a class I inhibitor located in the nucleus of the cell, where it can modulate inflammation in macrophages and monocytes by inhibiting the NFkB complex [93]. On the contrary, *HDAC5* is a class II inhibitor, which can migrate into the nucleus upon phosphorylation and mediate important anti-inflammatory functions [94]. Thalidomide and its derivatives, pomalidomide and lenalidomide, also share *HDAC2* as a molecular target. Thalidomide is an immunomodulatory agent and works by a number of mechanisms including the stimulation of T cells as well as decreasing TNF production. Importantly, these compounds also share anti-angiogenic properties and inhibit the proliferation of endothelial vascular cells [95].

Moreover, we identified proteasome inhibitors, sharing both antiviral and anti-angiogenic activity [96]. The ubiquitin–proteasome system plays an important role in virus replication and cell cycle, thus inhibiting virus entry, genome replication and viral protein synthesis. Proteasome inhibitors have already been pointed out as therapeutic strategies against other coronaviruses, since they can also limit the cytokine storm associated with the abnormal immunological response induced by the virus [97]. Most proteasome inhibitors can inhibit the NFkB-mediated production of IL-6, and, by inhibiting the NFkB transcription factor, they also exert an important anti-angiogenic effect [98]. Remarkably, *HDAC* inhibitors, proteasome inhibitors and thalidomide derivatives, are all currently used as a therapeutic regimen against multiple myeloma, an oncological condition in which myeloma cells produce a microenvironment enriched with pro-angiogenic factors, such as VEGF and IL-6 [95]. In conclusion, the four classes of drugs identified by the UKS share both immuno-modulatory and anti-angiogenic properties and are therefore good candidates in counteracting both the acute cytokine storm as well as endothelial and vascular complications.

## Conclusions

Characterizing the cascade of events taking place at multiple levels in response to SARS-CoV-2 infection is urgently needed as the COVID-19 pandemic keeps rampaging worldwide. Here, we interrogated a unified network of public biomedical data, the Unified Knowledge Space (UKS), in order to elucidate the molecular alterations characterizing the SARS-CoV-2 infection.

By assuming that early viral responses are mediated by virus-interacting genes, while the downstream effects of infection are mediated by genes whose expression is altered, we interrogated the UKS in search of a novel set of intermediate genes that would help to further characterize the COVID-19 pathogenesis. Our analysis highlighted genes representing functions related to fibrosis and vascular remodelling, implying further long-term consequences of SARS-CoV-2 infection. Furthermore, we identified a set of drugs with at least one target present in each of the identified gene sets: proteins known to interact with SARS-CoV-2 (PI, as defined by Gordon *et al.* [8]), differentially expressed (DE) genes in multiple biological systems infected by SARS-CoV-2 (Blanco-Melo *et al.* [9]) and intermediate genes (IN, newly discovered here). Our results point to therapeutic classes with immunomodulatory and anti-angiogenic roles.

In conclusion, the robust network-based approach applied here helps to shed light on the details of the SARS-CoV-2–host interaction, suggesting possible long-term effects of the viral infections, and highlights important therapeutic targets, paving the way to new drug repositioning studies. Furthermore, due to the high flexibility of the UKS, our strategy can be applied to study the molecular alterations induced by other diseases or by the exposure to drugs or chemicals.

**Key Points**

- Integrated molecular network analysis can help to clarify the pathogenesis of complex diseases and suggest novel drug targets.
- By mapping SARS-CoV-2 first physical interactors and COVID-19 downstream differentially expressed genes on the integrated human molecular network, we identified a new set of intermediate genes.
- The newly discovered set of intermediate genes underlies important aspects of COVID-19 pathogenesis and long-term consequences, pointing to lung tissue remodelling and fibrosis.
- We highlighted immuno-modulatory and anti-angiogenic drugs targeting multiple genes in each and every relevant set: physical interactors, intermediate and downstream effectors.

## Supplementary Material

Supplementary_file_S1_bbaa417

Supplementary_file_S2_A549_bbaa417

Supplementary_file_S3_biopsies_bbaa417

Supplementary_file_S4_Calu3_bbaa417

Supplementary_file_S5_NHBE_bbaa417

Supplementary_file_S6_bbaa417

Supplementary_file_S7_bbaa417

## Notes

**Alisa Pavel** is a Ph.D. student in Prof. Dario Greco’s group at Tampere University. She holds a M.Sc. in Computer Science (2019) from the University of Edinburgh. Her areas of expertise are biological network modelling and their analysis, as well as big data modeling for toxicogenomics and pharmacology.

**Giusy del Giudice** is Ph.D. student in Prof. Dario Greco’s group at the Tampere University. She holds a M.Sc. in Medical Biotechnology (2019). Her areas of expertise are the analysis and modelling of toxicogenomics and pharmacogenomics data, and systems immunology.

**Dr. Antonio Federico** works as a postdoctoral researcher in Prof. Dario Greco’s group at the University of Tampere. Dr. Federico holds a master degree in Molecular Biology (2014) and an international PhD with a dissertation about multi-layer integration of molecular networks to uncover the mechanisms of drug sensitivity of cancer-related genes for the development of tailored pharmacological therapies (2019). His research interests lie in network analysis, predictive pharmacology, systems biology and multi-omics data analysis and integration.

**Antonio Di Lieto** is MD, Ph.D. specialist in psychiatry. He obtained his Ph.D. in Behavioural science and learning processes. He has previously researched the biological correlates of psychiatric syndromes and molecular pharmacology. He is currently working as senior consultant psychiatrist at the Department of Forensic Psychiatry of the University of Aarhus, Denmark.

**Dr. Pia A.S. Kinaret** is senior postdoctoral researcher in Prof. Dario Greco’s group at the University of Helsinki. Kinaret has degrees in biotechnology (2009) and bioinformatics (2012), and she obtained the Ph.D. in genetics (2017). Her current research focuses on immunotoxic effects and immunomodulatory potential of manufactured nanomaterials. In her work she utilizes *in vivo*, *in vitro* and systems biology approaches. She currently receives funding from Orion Research Foundation sr.

**Dr. Angela Serra** is a senior postdoctoral researcher in Prof. Dario Greco’s group at the University of Tampere. Serra has a master’s degree in Computer Science (2013), and she obtained the Ph.D. in Computer Science and Information Engineering with a dissertation on multi-view learning and data integration approaches for multi-omics data (2017). Her current research focuses on data modelling, machine learning, network inference, data integration for toxicogenomics, bioinformatics and cheminformatics.

**Prof. Dario Greco** is professor of bioinformatics at the Faculty of Medicine and Health Technology, Tampere University and principal investigator at the Institute of Biotechnology, University of Helsinki, FInland. To date, he authored over 130 articles in peer reviewed journals in the areas of nanotoxicology, systems toxicology, toxicogenomics, predictive toxicology, data modelling and bioinformatics. He currently receives fundings from the Academy of Finland, Business Finland and the EU (H2020 and IMI2 programs).
