# Supplementary material for: Integrated network analysis reveals new genes suggesting COVID-19 chronic effects and treatment
Source: Brief Bioinform. 2021 Feb 11;22(2):1430–41. doi: 10.1093/bib/bbaa417 (PMC7929418; doi:10.1093/bib/bbaa417)
Supplement: Supplementary_file_S1_bbaa417 [file supplementary_file_s1_bbaa417.pdf]

```
#create Unified Knowledge Space

#download human Ensembl gene Ids and gene names

genes = "http://www.ensembl.org/biomart/martview"

#download PPI data

Hippie = download "http://cbdm-01.zdv.uni-mainz.de/~mschaefer/hippie/
hippie_current.txt"

HitPredict = download "https://ndownloader.figshare.com/files/24131579"

String = download "https://stringdb-static.org/download/
protein.links.v11.0/9606.protein.links.v11.0.txt.gz"

KEGG = download via bioservices API (https://bioservices.readthedocs.io/en/master/
kegg_tutorial.html)

#download drug data

DrugBank = "https://go.drugbank.com/releases/latest"

OpenTarget = "https://storage.googleapis.com/open-targets-data-releases/19.02/output/
19.02_association_data.json.gz"

#add to UKS

for gene in genes:

    add gene as GENE with Ensembl_ID = gene.Ensembl_ID and gene_symbol =
gene.gene_name to UKS


for db in [Hippie, HitPredict, String, Kegg]:

    for  relationship in db: # relationship is list of two interaction gene ids

        add "interaction" between  relationship[0] and  relationship[1] and add db to
interaction.source to UKS

for db in [DrugBank, OpenTarget]:

    for relationship in db: # relationship is list of drug and its gene target id

        add  relationship[0] as DRUG to UKS #add drug to UKS

        add "targets" between  relationship[0] and  relationship[1] with
targets.source = db to UKS

#extract robust gene - gene network from UKS
network = from UKS get "interaction" where length(interaction.source) >= 3

#preprocess transcriptomics data (to generate DE gene set)

transcriptomics = download "https://www.ncbi.nlm.nih.gov/geo/query/acc.cgi?
acc=GSE147507"

filtered = filter low read counts from transcriptomics

normalized = normalize filtered

de = retrieve differential expressed genes from normalized
```

```
# retrieve PI gene set
pi = download "https://static-content.springer.com/esm/
art%3A10.1038%2Fs41586-020-2286-9/MediaObjects/41586_2020_2286_MOESM6_ESM.xlsx"

#find all shortest paths between all de and pi gene pairs on network
paths = []
for g1 in de:
    for g2 in pi:
        path = shortest_path(network, source=g2, target=g1, algorithm="dijkstra")
#path is list of genes visited including source and target
        paths.append(path)

#get counts for each gene (if not in de or pi) in paths if path length is > 2 to have
at least one intermediate gene
gene_counts = {} #saves how often each gene occures as an intermediate gene
for path in paths:
    if length(path) > 1:
        for gene in path:
            if gene is not first or last in path:
                add occurence of gene to gene_counts

#generate background distribution for hypergeometric test
all_paths = shortest_path(network, algorithm="dijkstra") #all shortest paths between
all nodes in network
all_gene_counts = {} #saves how often each gene occures as intermediate gene on all
shortest paths in the network
for path in all_paths:
    if length(path) > 1:
        for gene in path:
            if gene is not first or last in path:
                add occurence of gene to all_gene_counts


#identify statistical significant intermediate genes
in = adjust_pvalues(hypergeometric_test(gene_counts, all_gene_counts)) where
adjusted_pvalues <= 0.05

#functional enrichment for de, pi & in
pathways_de = enriched_pathways(de)
pathways_pi = enriched_pathways(pi)
pathways_in = enriched_pathways(in)

compare pathways_de, pathways_pi, pathways_in

#retrieve drugs targeting de, pi and in from UKS
de_drugs = from UKS get drugs where DRUG targets gene in de
pi_drugs = from UKS get drugs where DRUG targets gene in pi
in_durgs = from UKS get drugs where DRUG targets gene in in

#get intersection
interessting_drugs = intersection(de_drugs, pi_drugs, in_drugs)

map interessting_drugs to functional drug group
```
